# 102, Danysz Compound

**Published:** 1916-09

**Authors:** 


### ABSTRACTS.

---

**102, Danysz Compound.** Roger, Dalimier and Frenkel, Bull. De l'Acad. de Sci. Mch. 20, report favorably on this substitute for salvarsan. It contains arsenic, antimony and silver.

---

Other abstracts fill out pages at end of original articles and in advertising section.

